# The Effect of Dietary Oil Level and Multi‐Enzyme Supplementation on Performance, Bone Mechanical Properties and Mineral Contents, Intestine Morphology and Immune Response in Broiler Chickens

**DOI:** 10.1002/vms3.70246

**Published:** 2025-04-02

**Authors:** Avisa Akhavan Khaleghi, Abolghasem Golian, Hassan Nasiri Moghaddam, Heydar Zarghi

**Affiliations:** ^1^ Department of Animal Science Faculty of Agriculture Ferdowsi University of Mashhad Mashhad Iran

**Keywords:** broilers, non‐starch polysaccharide multi‐enzyme, performance, soy oil, wheat

## Abstract

**Background:**

Supplementing broiler diets with non‐starch polysaccharide multi‐enzymes (NMEs) has been shown to improve nutrient utilization and performance. However, the interaction between dietary soybean oil levels and NME supplementation in diets requires further exploration.

**Objectives:**

The aim of the present study was to evaluate the dietary soya bean oil level and NME supplementation in a low‐energy wheat‐based diet's effects on performance, bone mechanical properties and mineral contents, blood metabolites, small intestine morphology and immunity criteria in the broiler chickens.

**Methods:**

A total of 360 one‐day‐old mixed‐sex Ross 308 broiler chicks were randomly assigned to 6 treatments, 5 replicates/treatment and 12 (6 females and 6 males) birds/replicate. Experimental treatments were included in a factorial arrangement of 0%, 6% and 12% levels of diet metabolizable energy supplied by soy oil (MESO) with/without NME supplementation. For starter (1–10 days), grower (11–24 days) and finisher (25–38 days) rearing periods, six isocaloric and isonitrogenous experimental diets were formulated and fed ad libitum.

**Results:**

There was no significant difference in growth performance traits between birds fed diets with different levels of MESO during the starter period. As well as increased dietary MESO levels, weight gain and feed conversion ratio during grower, finisher and whole rearing periods linearly improved. Moreover, abdominal fat relative weight, breast meat cooking lost and jejunum muscular thickness linearly increased, and liver relative weight and jejunum crypt depth linearly decreased. Dietary NME supplementation led to improved production performance during the starter, grower and whole experimental periods; enhanced tibia bone strength and abdominal fat; decreased gizzard and intestine relative weight; and decreased jejunum muscular thickness.

**Conclusions:**

It was concluded that dietary NME supplementation and supply of 12% of broiler chickens metabolizable energy requirements through soy oil have a positive effect on the growth performance, bone strength, liver and small intestine health of broiler chickens fed low‐energy wheat‐based diet.

## Graphical abstract

Dietary supplementation by vegetable oil (VO) plays an important role in lipid metabolism and its body deposition. Lower liver relative weight of broiler chickens fed a diet supplying 12% of ME requirement through VO suggests that VO could cause an inhibition of lipogenesis.

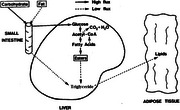

## 1 Introduction

Cereal's grain provides edible starch, and a variety of them can be utilized in poultry diets as an energy source. Corn contains much energy, so it is regularly used in commercial poultry diets (Ebrahimi et al. 2017). The future consequence of the rising demand for corn would be a continuously rising price (Salari, Golian, and Hassanabadi 2024). Additionally, due to limitations in corn production in Iran, a substantial portion must be imported to meet the demand (Chizari and Hajiheidary 2010). Wheat can be cultivated in most regions of the country, leading to its common use in poultry feed (Hossaninejad, Zarghi, and Golian 2021). Moreover, it is common to use wheat in many countries (Australia and New Zealand) as the key energy source in poultry diets (Zarghi et al. 2022). In the cell walls of wheat and certain other cereals, such as rye, triticale and barley, there are significant amounts of soluble non‐starch polysaccharides (NSP), particularly xylans and arabinoxylans. An increase in digest viscosity, a reduction in the physical mixing of intestinal contents and diminished nutrient bioavailability due to decreased movement of components in the gastrointestinal tract (GIT) are actually the results of high levels of those compounds in poultry diets (Attia et al. 2019). A significant improvement in weight gain (WG) and feed efficiency was reported for broilers (Teymouri, Zarghi, and Golian 2018), turkeys (Zarghi, Golian, Aghel et al. 2010) and quails (Ebrahimi et al. 2017) when viscose cereal–based diets were supplemented with exogenous enzyme compounds that break down plant cell walls.

Vegetable oils (VOs) are energy‐dense ingredients for poultry, and these oils are typically used to create high‐energy diets (Salari, Golian, and Hassanabadi 2024) because their energy content is mostly thrice as high as that of other feedstuffs (NRC 1994). Improved feed texture, enhanced palatability and decreased dustiness are further benefits of the added VO in the poultry diet (Tancharoenrat et al. 2014). If the price is reasonable against the cereal grains, adding 20–50 g/kg of VO to poultry diets is recommended (Richard et al. 2010). Digestion and absorption of lipids depend on various factors, such as fat type, diet composition and age of the bird (Tancharoenrat et al. 2013). Consequently, feed additives that improve the digestion and absorption of lipids could be used in diets to offer more energy per unit of feed volume, and also enzyme supplementation is of this type, especially in feeding viscose cereal–based diets situation.

By adding VO, we can achieve both supplementation of calories and improvement of carcass characteristics (Azman et al. 2004; Fébel et al. 2008). Notably, the level at which we supply the VO in diets influences the composition of the fatty acid of the broiler carcass. Adipose tissues incorporated directly by dietary fatty acids are the reason for the detected changes of fatty acids in broiler tissues. Fatty acids are also known as modulators of immune responses (Fritsche, Cassity, and Huang 1991; Nayebpor, Hashemi, and Farhomand 2007). Nayebpor, Hashemi, and Farhomand (2007) reported that increasing the dietary levels of soybean oil could result in improvement of antibody titres against infectious bursal disease virus. The current experiment has been conducted to study the growth performance, visceral organ characteristics, blood metabolites, bone quality, intestine morphology and immunity of broiler chickens fed a low‐energy wheat‐based diet, which contains different levels of dietary metabolizable energy supplied by soy oil (MESO) supplemented with and without non‐starch polysaccharide multi‐enzyme (NME).

## 2 Materials and Methods

### 2.1 Birds, Housing and Care

A total of 360 one‐day‐old mixed‐sex Ross 308 broiler chicks were supplied from a local commercial hatchery. Chicks were randomly assigned to 30 pens, 6 dietary treatments with 5 replicates and 12 (6 females and 6 males) birds each. Each pen was 1 m^2^ (100 × 100 × 70 cm^3^ in size; *L* × *W* × *H*), covered with wood shavings, and equipped with a hanging pan feeder (7 cm length/bird) placed in the middle section of each pen and nipple drinkers (2 nipples/pen) at the front. The ambient temperature was maintained at 32°C ± 2°C for 3 days and then decreased by 0.5°C per day to reach 20°C–22°C and after that was kept constant. The relative humidity was maintained at 50%–60%. Continuous lighting was provided for the first 3 days, followed by an 18:6 h light:dark for the remainder of the experimental period. Throughout the experiment, birds had unrestricted access to feed and fresh water.

### 2.2 Experimental Design and Diets

The experiment was done in a completely randomized design (CRD) with a factorial arrangement of three levels of soy oil replaced for supplying 0%, 6% and 12% of diet metabolizable energy (MESO) with/without NME supplementation in a low‐energy wheat‐based diet. The dietary NME supplementation levels were 0 and 200 mg/kg of diet of an enzyme cocktail (Endopower β, Union Center—Seoul—Korea, containing α‐galactosidase 35 U/g, galactomannanase 110 U/g, xylanase 1500 U/g and β‐glucanase 1100 U/g). To formulate the experimental diets, the nutrient recommendations of Ross 308 manual for broiler chickens were followed as a basis (Aviagen 2022). Three basal diets were formulated for starter (1–10 days), grower (11–24 days) and finisher (25–38 days) rearing periods (Table 1) in a minimum cost equation by user‐friendly feed formulation done again software, University of Georgia, Athens, GA, USA (UFFDA 1992). Each of the diets was divided into two equal portions, and the NME in replacement with corn starch (0 and 200 mg/kg) was added to the top of each portion and mixed well to make six experimental diets for each period of the experiment.

**TABLE 1:** Ingredients and nutrients composition of the experimental diets.^a^

Levels of dietary metabolizable energy supply by soy oil (MESO), %								
Starter (Days 1–10)			Grower (Days 11–24)			Finisher (Days 25–38)		
Items	0	6	12	0	6	12	0	6	12
**Ingredients, g/100 g as‐fed basis**									
Wheat	64.57	57.54	50.52	71.16	63.95	56.75	78.77	71.44	64.11
Soy meal	30.30	32.56	34.81	24.16	26.47	28.79	16.88	19.28	21.64
Soy oil	—	2.03	4.06	—	2.08	4.16	—	2.12	4.23
DCP	1.44	1.49	1.54	1.16	1.22	1.27	0.92	0.96	1.03
Limestone	1.32	1.32	1.25	1.26	1.23	1.20	1.20	1.16	1.13
Common salt	0.33	0.34	0.35	0.33	0.33	0.35	0.31	0.32	0.34
l‐Lysine hydrochloride	0.35	0.33	0.30	0.32	0.29	0.26	0.34	0.29	0.26
dl‐Met	0.38	0.37	0.37	0.33	0.33	0.33	0.31	0.31	0.31
l‐Thr	0.19	0.19	0.18	0.16	0.15	0.14	0.15	0.14	0.14
Vitamin premix^b^	0.25	0.25	0.25	0.25	0.25	0.25	0.25	0.25	0.25
Mineral premix^c^	0.25	0.25	0.25	0.25	0.25	0.25	0.25	0.25	0.25
Corn starch	0.12	0.12	0.12	0.12	0.12	0.12	0.12	0.12	0.12
Sand	0.50	3.25	6.00	0.50	3.33	6.15	0.50	3.35	6.21
**Determined nutrients composition** ^d^ **, as‐fed basis**									
ME, kcal/kg	2830	2830	2830	2890	2890	2890	2950	2950	2950
CP (%)	21.85	21.85	21.85	20.04	20.04	20.04	17.98	17.98	17.98
Calcium (%)	0.91	0.91	0.91	0.81	0.81	0.81	0.73	0.73	0.73
Available phosphorus (%)	0.46	0.46	0.46	0.41	0.41	0.41	0.36	0.36	0.36
Sodium (%)	0.20	0.20	0.20	0.20	0.20	0.20	0.20	0.20	0.20
Digestible lysine (%)	1.22	1.22	1.22	1.08	1.08	1.08	0.95	0.95	0.95
Digestible threonine (%)	0.82	0.82	0.82	0.72	0.72	0.72	0.64	0.64	0.64
Digestible methionine (%)	0.63	0.63	0.63	0.44	0.44	0.44	0.51	0.51	0.51
Digestible SAA (%)	0.90	0.90	0.90	0.81	0.81	0.81	0.74	0.74	0.74

Abbreviations: CP, crude protein; ME, metabolizable energy; SAA, sulphur amino acids.

^a^ Each of the above diets was divided into two equal portions, and the non‐starch polysaccharide multi‐enzyme (NME) (Endopower GNC Bioferm‐Canada, containing alpha‐galactosidase 35, galactomannanase 110, xylanase 1500 and beta‐glucanase 1100 U/g) in replacement with corn starch at the rate of 0 and 200 mg/kg of diet was added to the top of each portion and mixed well to make six experimental diets for each one of the age periods.

^b^ Vitamin premix supplied per kilogram diet: vitamin A (trans‐retinyl acetate), 12,500 International units; vitamin D3 (cholecalciferol), 5000 International units; vitamin E (tocopheryl acetate), 80 mg; vitamin K3, 3.2 mg; thiamine, 3.2 mg; riboflavin, 8.6 mg; panthothenic acid, 8.6 mg; pyridoxine, 4.86 mg; B12 cyanocobalamin, 0.02 mg; niacin, 62.51 mg; biotin, 0.25 mg; folic acid, 2.2 mg; antioxidant 2.5 mg.

^c^ Mineral premix supplied per kilogram diet: Fe, 20.23 mg; Mn, 120 mg; Zn, 110 mg; Cu, 16 mg; I, 1.252 mg; Se, 0.3 mg. Choline chloride, 300 mg.

^d^ The determined ingredient analysis data were used to calculate nutrient composition, crude protein, calcium and sodium measured by the AOAC (2002) methods; metabolizable energy, digestible amino acids, and available phosphorus were measured by the NIR method.

### 2.3 Data Collection and Sampling

#### 2.3.1 Growth Performance

The birds of each experimental unit (pen) were weighed at 10, 24 and 38 days of age. In order to minimize error due to digestive contents, the birds were starved for 4 h before weighing. The feed intake (FI) of each experimental unit was determined by subtracting the quantity of feed remaining at the end from the sum total feed provided throughout each rearing period and data adjusted for mortality. The growth performance traits include average live body weight (LBW) at 10, 24 and 38 days of age, FI, WG and feed conversion ratio (FCR) during the starter, grower, finisher and whole rearing periods.

#### 2.3.2 Tibia Bone Mechanical Properties and Mineral Contents

At 28 days of age, one (5/treatment) male bird that was near to the average pen male chicks’ weight was randomly selected, weighted and euthanized by cervical dislocation. Immediately after slaughter, every tibia bone was cut up, and any adhering tissue was separated away from it, and it was inserted in a sealed plastic bag and maintained at −20°C for further examination. In the following steps of analysis, the right tibia bone was subjected to osteometric measurements and strength tests, whereas the left tibia bone was used for mineral content analysis.

##### 2.3.2.1 Osteometric Measurements

After overnight thawing, the right tibia bone length and width were determined by digital calliper (0.05 mm, Model 1116‐150, Insize Co. Ltd., Suzhou, China), and then it was weighed by a digital electronic scale (0.001 g, Model GF 400, A&D Weighing Co. Ltd., CA, USA). The weight of each bone was determined when immersed in distilled water to calculate specific gravity (SG) by Archimedes method and by using the following formula (Hempe, Laukxen, and Savage 1988):

$$\begin{array}{ccc} & & \;\mathrm{Bone}\;\mathrm{specific}\;\mathrm{gravity},\;g/cm^{3}\\ & & \;=\frac{\mathrm{Bone}\;\mathrm{weight}}{\left(\mathrm{Bone}\;\mathrm{wight}-\mathrm{Bone}\;\mathrm{weight}\;\mathrm{immersed}\;\mathrm{in}\;\mathrm{water}\right)} \end{array}$$

##### 2.3.2.2 Mechanical Properties

Using the three‐point bending test method, the mechanical properties of bones were determined, performed on an Instron Universal Testing Machine (Model H5KS, Tinius Olsen Company). The support span was 40% of the bone length (Muszyński et al. 2018). The bone was loaded in the anterior–posterior plane, and a perpendicular load cell was used to apply loading to the midpoint with a displacement rate of 5 mm/min until fracture (Cufadar, Olgun, and Yildiz 2011). The results were recorded as the force (N) required to reach the structural failure of the tibia, stiffness (N/mm) and energy (J) between each bone.

##### 2.3.2.3 Mineral Contents

The left tibia bones were taken away from the freezer and located on a countertop for thawing. Later, they were weighed and dried at 55°C for 72 h in a forced‐ventilation oven. After that, the samples were taken out and defatted in a Soxhlet extractor for 8 h; then they were put back in the forced ventilation oven at 55°C for 72 h and were weighed again to determine the dry matter (Xavier et al. 2015). After weighing, bones were ground to determine bone ash and mineral contents, and then they were put in a muffle furnace at 600°C for 16 h (Kolakshyapati et al. 2019). After the addition of weighted bone ash (0.1 g) to concentrated (65%) nitric acid (5 mL), the samples were predigested for a period of 100 min at 80°C in a bain‐marie. After digestion, the digested solution was combined with 20 mL of high‐purity deionized water, and the solution was quantitatively passed through filter paper, transferred to a 50 mL container and adjusted for volume with high‐purity deionized water (Kolakshyapati et al. 2019). Analysis for Ca, phosphorus, Mg, Mn and Zn was carried out using an inductively coupled plasma optical emission spectrometer (ICP‐OES; Agilent Australia, Victoria, Australia).

#### 2.3.3 Intestinal Morphology

At 28 days of age, one (5/treatment) male bird that was near to the average pen male chicks’ weight was randomly selected, weighted and euthanized by cervical dislocation. Immediately after slaughter, a portion (0.5 cm in length) of the jejunum segment midpoint was taken, flushed by 0.9% saline and fixed in 10% neutral buffered formalin solution until histological work. In a histology lab, the tissue samples were dehydrated by ethanol, cleared in xylene, infiltrated with paraffin and embedded in wax blocks. The embedded tissue samples were sectioned (5 µm thickness) and then stained with haematoxylin and eosin (H&E).

Morphological measurements of intestinal slides were performed by using an Olympus Light Microscope (Model U‐TV0.5 XC‐2, Olympus Corporation, BX41, Olympus, Tokyo, Japan) and Image‐Pro Plus V 4.5 software package on nine villi chosen from each slide, and only vertically oriented villi were selected for measuring (Zarghi et al. 2022). The morphological traits were (1) villus height (distance from the tip of the villi to the crypt junction), (2) villus width (average of villus width at one‐third and two‐thirds of villus length), (3) crypt depth (CD) (distance from the base of the villi to the submucosa) and (4) muscular thickness. The villus surface area was calculated according to the following formula (Ebrahimi et al. 2017):

$$VSA\;\mu m^{2}=\frac{1}{2}VW\times VH\times 2\times \pi$$

where VSA is the villus surface area; VW is the villus width; VH is the villus height, and *π* = 3.14.

#### 2.3.4 Carcass Characteristic

At 38 days of age, one (5/treatment) male bird that was near to the average pen male chicks’ weight was randomly selected and, after 4 h of starvation, was weighed and slaughtered. Edible carcass parts, abdominal fat pad (including fat surrounding gizzard, bursa of Fabricius, cloaca and adjacent muscles), digestive tract organs and visceral organs were dissected and weighed (0.001‐g, model GF 400; A&D Weighing, San Jose, CA, USA) individually.

##### 2.3.4.1 Cooking Loss (CL)

Femur and breast meat CL was measured as proportionate weight loss of a meat sample after cooking at 70°C for 40 min (Nikbakhtzade, Zarghi, and Golian 2024).

#### 2.3.5 Humoral Immune Response

To evaluate the humoral immune response of broiler chickens, sheep red blood cells (SRBCs), as a non‐pathogenic antigen, were used. Two (10/treatment) male birds from each replicate were injected with 0.5 mL of 5% SRBC suspension into the breast muscle at 23 and 30 days of age, and blood samples were collected 7 days after the injection. Later, the microhaemagglutination activity (HA) of serum was estimated, and the antibody concentration (log2) was measured, following the standard procedure (Allahdo et al. 2018).

#### 2.3.6 Blood Metabolites

At 38 days old, one (5/treatment) male bird was randomly selected, and the blood samples were gathered from the right‐wing vein into non‐heparinized tubes. After allowing for the completion of clotting, blood samples were centrifuged at 1900 *g* for 10 min at 4°C to extract serum. Subsequently, serum samples were frozen at −20°C for later analysis. Before testing, samples were removed from the freezer, thawed and warmed to room temperature (Zarghi et al. 2022). In the blood samples, the calcium, phosphorus, triglyceride (TG), total cholesterol (TC), high‐density lipoprotein cholesterol (HDL‐C), total fat (TF), uric acid (UA), creatinine, total protein (TP), albumin, alanine aminotransferase (ALT), aspartate aminotransferase (AST) and alkaline phosphatase (ALP) concentrations were measured with a multi‐test automated random‐access system auto‐analyser (Cobas Bio, Roche, Basel, Switzerland) with kits from Pars Azmoon Company, Iran. The following formula was used for low‐density lipoprotein cholesterol (LDL)‐C calculation (Amer et al. 2020):

$$\mathrm{LDL}-C\;=\frac{\mathrm{TC}}{1.19}+\frac{\mathrm{TG}}{1.9}-\;\frac{\mathrm{HDL}}{1.1}-38$$

where LDL‐C is the low‐density lipoprotein cholesterol; TC is the total cholesterol; TG is the triglyceride; and HDL is the high‐density lipoprotein cholesterol.

### 2.4 Statistical Analysis

The results of the experiment were analysed in a CRD with the factorial arrangement, using statistical software SAS 9.4 general linear model (GLM) procedure (SAS 2014). Mean comparison was done by Tukey's test. The results were considered statistically significant when *p* < 0.05. The linear and quadratic responses to dietary MESO levels were calculated by using polynomial orthogonal contrasts. Statistical plan model is shown in the following formula:

$$Y_{ij}=\mu +\alpha_{i}+\beta_{j}+{\left(\alpha \beta \right)}_{ij}+\varepsilon_{ij}$$

where *Y_ij_* is the value that view, *μ* is the mean population, *α_i_* is the effect of dietary MESO levels, *β_j_* is the effect of dietary NME supplementation, (*αβ*)*_ij_* is the interaction effects and *ε_ijk_* is the effect of experimental error.

## 3 Results and Discussion

### 3.1 Growth Performance

The average LBW of 1‐day‐old chicks was about 44 ± 2.07 g/b, and chicks pen weights were similar before they were allocated to the dietary treatments. The average LBW, WG, FI and FCR of chicks fed diets with different levels of MESO and supplemented with/without NME during starter (1–10 days), grower (11–24 days), finisher (25–38 days) and whole (1–38 days) experimental periods are shown in Table 2. FI during any or the whole period of the experiment, LBW at 10 days of age, WG during the starter period and FCR during the starter and grower periods were not influenced by MESO level. However, LBW at 24 and 38 days of age (*p* < 0.01), WG during the grower (*p* < 0.01), finisher (*p *< 0.05) and whole (*p* < 0.01) experimental periods and FCR during the finisher and whole (1–38 days) experimental periods (*p* < 0.01) were significant and linearly improved by increasing dietary MESO levels. The 24‐ and 38‐day LBW, WG during the grower, finisher and whole experimental periods in the birds fed a diet with 12% MESO were 8.70%, 12.57%, 11.95%, 16.69% and 11.47% higher than those fed wheat‐soy diet (0% MESO), respectively. Similarly, during the above periods, FCR was improved by 7.61%, 19.40% and 13.27% in the birds fed with a diet with 12% MESO, as compared to those fed with wheat‐soy (0% MESO) diet. The NME addition significantly improved 10‐, 24‐ and 38‐day LBWs (*p* < 0.01), WG during the starter and whole experimental periods (*p* < 0.01), and FCR during the grower and whole experimental periods (*p* < 0.01). FI during any and/or all of the experiment periods was not significantly influenced by NME supplementation. The interaction between dietary MESO level and NME supplementation on growth performance traits was not significant.

**TABLE 2:** Effect of dietary non‐starch polysaccharide multi‐enzyme (NME) supplementation and levels of metabolizable energy supply by soy oil (MESO) on growth performance of broiler chickens fed wheat‐based diet.^1^

		Live body weight, g/b				Feed intake, g/d/d				Weight gain, g/d/d				Feed conversion ratio			
NME, mg/kg	MESO level^2^, (%)	Day 1	Day 10	Day 24	Day 38	Days 1–10	Days 11–24	Days 25–38	Days 1–38	Days 1–10	Days 11–24	Days 25–38	Days 1–38	Days 1–10	Days 11–24	Days 25–38	Days 1–38
0	0	44.0	279^c^	826^b^	1609^c^	23.19	59.11	96.18	63.31	23.50^b^	39.08^b^	55.90	41.18^c^	0.987	1.512^a^	1.739^a^	1.546^a^
	6	43.0	280^bc^	867^b^	1756^abc^	23.25	59.60	92.39	62.11	23.72^b^	41.90^ab^	63.52	45.08^abc^	0.982	1.427^ab^	1.465^ab^	1.379^ab^
	12	44.9	284^bc^	871^b^	1809^abc^	22.17	60.14	90.68	61.40	23.91^b^	41.91^ab^	67.03	46.43^abc^	0.931	1.440^ab^	1.382^ab^	1.330^b^
200	0	43.5	294^abc^	855^b^	1683^bc^	23.35	55.92	89.79	59.83	25.04^ab^	40.07^b^	59.18	43.15^bc^	0.932	1.400^ab^	1.534^ab^	1.392^ab^
	6	44.0	309^a^	897^ab^	1871^ab^	23.39	56.34	89.62	59.93	26.48^a^	41.98^ab^	69.64	48.09^ab^	0.883	1.345^ab^	1.293^b^	1.248^b^
	12	45.1	302^ab^	955^a^	1897^a^	24.46	59.09	90.17	61.43	25.64^ab^	46.71^a^	67.27	48.73^a^	0.954	1.267^b^	1.352^b^	1.263^b^
SEM		0.85	4.99	18.15	47.25	0.68	2.14	2.04	1.31	0.49	1.38	3.48	1.23	0.029	0.052	0.085	0.050
	0	43.7	286	840^b^	1646^b^	23.27	57.51	92.98	61.57	24.27	39.58^b^	57.54^b^	42.17^b^	0.959	1.456	1.642^a^	1.468^a^
	6	43.5	295	882^ab^	1814^a^	23.32	57.97	91.01	61.02	25.10	41.94^ab^	66.58^a^	46.59^a^	0.933	1.386	1.379^b^	1.313^b^
	12	45.0	293	913^a^	1853^a^	23.31	59.61	90.43	61.41	24.78	44.31^a^	67.14^a^	47.58^a^	0.943	1.353	1.368^b^	1.296^b^
SEM		0.61	3.53	12.84	33.41	0.48	1.51	1.44	0.92	0.35	0.98	2.46	0.88	0.020	0.037	0.060	0.035
0		43.9	281^b^	855^b^	1725^b^	22.87	59.61	93.08	62.28	23.71^b^	40.97	62.15	44.23^b^	0.967	1.460^a^	1.532	1.418^a^
200		44.2	301^a^	902^a^	1817^a^	23.73	57.12	89.86	60.40	25.72^a^	42.92	65.36	46.66^a^	0.923	1.337^b^	1.394	1.301^b^
SEM		0.49	2.88	10.48	27.28	0.39	1.23	1.18	0.75	0.28	0.80	2.01	0.72	0.017	0.030	0.049	0.029
Source of variation, *p* value																	
MESO level		0.258	0.251	0.002	0.001	0.997	0.594	0.434	0.912	0.253	0.008	0.018	0.001	0.652	0.152	0.005	0.003
NME		0.706	<.001	0.004	0.025	0.132	0.165	0.065	0.091	<.001	0.097	0.271	0.024	0.076	0.008	0.059	0.008
Interaction		0.842	0.360	0.232	0.907	0.216	0.843	0.363	0.412	0.417	0.215	0.704	0.913	0.123	0.682	0.549	0.667
Regression analysis in responses to MESO level^b^																	
Liner		0.590	0.303	0.002	0.015	0.952	0.329	0.243	0.906	0.457	0.003	0.040	0.014	0.599	0.085	0.022	0.031
Quadratic		0.329	0.417	0.802	0.148	0.962	0.748	0.701	0.693	0.332	0.999	0.164	0.141	0.508	0.709	0.114	0.156

*Note*: Means within each column for each effect with uncommon superscripts (a–c) differ significantly (*p*< 0.05).

^1^ The values are means of 15, 10 and 5 replicates for enzyme, oil level and interaction effect, respectively.

^2^ The level of dietary metabolizable energy supply by oil.

The current study findings are agreed with by other researchers who have mentioned that VO levels in the isocaloric and isonitrogenous diets did not affect the growth performance during the starter period in the broilers (Wongsuthavas et al. 2007). In the younger age, the capability for lipid digestion and absorption is not fully developed. The bile secretion is considered the first limiting factor, and after that, secretion of lipase, fatty acid binding protein (FABP) synthesis or other physiological factors could be the next limiting (Ravindran et al. 2016). It has also been reported that the synthesis of FABP is not sufficient in very young birds but is increased step by step until 4 weeks of age (Katongole and March 1980). The activities of all pancreatic enzymes (when they are expressed as units of activity per kilogram of body weight) are increased with age, while reaching a maximum on Day 8 for the lipase (Ravindran et al. 2016). In agreement with the results obtained from the present study, other researchers (Velasco et al. 2010; Nikbakhtzade, Zarghi, and Golian 2024; Salari, Golian, and Hassanabadi 2024) reported that increased fat levels in the grower and finisher diet improved the growth performance of broilers. This is done by optimizing the absorption of other nutrients in the diet, which means that the birds can obtain more energy from the diet compared to the calculated metabolizable energy. The synergistic effect of fats with other nutrients in the diet has been proposed; a higher fat content in the diet improves nutrient digestibility (Cho et al. 2008; Mirshekar et al. 2013) and leads to better performance. Because the use of fat in the diet reduces the feed passage rate through the digestive system and thus increases the digestibility and absorption of nutrients (Nikbakhtzade, Zarghi, and Golian 2024). Moreover, the reduction of dust with higher levels of fat in the diet may improve feed efficiency (Salari, Golian, and Hassanabadi 2024). In the current study, NME supplementation growth performance significantly improved. In agreement with our findings, several studies reported that the addition of enzymes significantly improved the performance of broiler chickens fed viscose cereal–based diet (Józefiak et al. 2007; Zarghi, Golian, Kermanshahi et al. 2010).

### 3.2 Carcass and Cut‐Up Parts

The breast and thigh CL and relative weight as a percentage of LBW of carcass and cut parts, GIT and visceral organs of broiler chickens fed a wheat‐based diet with different levels of MESO and supplemented with/without NME slaughtered at 38 days of age are shown in Table 3. There were not significant differences in carcass yield and/or cut‐up parts of birds fed diets with different levels of MESO with/without NME supplementation when slaughtered at 38 days of age. Abdominal fat percentage weight and breast meat CL significantly and linearly increased, and liver relative weight decreased (*p* < 0.05) by increasing dietary MESO level. Enzyme cocktail supplementation led to a significant (*p* < 0.05) increase in abdominal fat relative weight and reduced empty small and large intestine and gizzard relative weight. The interaction between dietary MESO level and NME supplementation on carcass cut parts, GIT and visceral organs relative weight was not significant.

**TABLE 3:** Effect of dietary non‐starch polysaccharide multi‐enzyme (NME) supplementation and levels of metabolizable energy supply by soy oil (MESO) on carcass and cut parts, GIT and visceral organs relative to the weight of broiler chickens fed wheat‐based diet (38 days of age)^1^.

		Carcass cut parts ^3^, g/100 g LBW					GIT organs, g/100 g LBW					Visceral organs, g/100 g LBW				Cooking lost, %	
NME, mg/kg	MESO level^2^, %	Carcass	legs	Breast	frame	AF	PV	Giz.	Pan	SI	LI	BF	Spleen	Liver	Heart	Femur	Breast
0	0	60.46	20.24	19.11	21.11	1.06^b^	0.45^a^	1.86	0.27	3.08^a^	0.86	0.15	0.10	2.56^ab^	0.47	37.89	36.19^b^
	6	60.15	19.80	21.81	18.54	1.57^b^	0.41^ab^	1.73	0.27	2.87^ab^	0.71	0.15	0.11	2.47^ab^	0.46	38.04	40.20^ab^
	12	59.05	19.21	20.45	19.39	1.60^ab^	0.43^ab^	1.68	0.28	2.98^ab^	0.72	0.13	0.13	2.26^b^	0.45	39.61	44.31^a^
200	0	61.80	19.89	22.69	19.49	1.76^ab^	0.41^ab^	1.76	0.23	2.69^bc^	0.76	0.12	0.10	2.78^a^	0.42	38.08	37.90^b^
	6	60.88	20.15	21.39	19.34	1.61^ab^	0.34^b^	1.75	0.26	2.70^bc^	0.75	0.13	0.14	2.56^ab^	0.42	37.65	39.14^b^
	12	59.54	19.08	21.31	19.15	2.40^a^	0.39^ab^	1.73	0.23	2.49^c^	0.77	0.10	0.12	2.29^b^	0.41	38.07	40.78^ab^
SEM		0.84	0.38	0.84	0.71	0.18	0.02	0.05	0.02	0.08	0.04	0.02	0.02	0.09	0.02	1.73	1.13
	0	61.18	20.06	20.90	20.30	1.41^b^	0.43^a^	1.81	0.25	2.88	0.81	0.13	0.10	2.67^a^	0.44	37.99	37.05^b^
	6	60.52	19.97	21.60	18.94	1.59^ab^	0.38^b^	1.74	0.26	2.78	0.73	0.14	0.12	2.52^a^	0.44	37.85	39.67^ab^
	12	59.30	19.15	20.88	19.27	2.00^a^	0.41^ab^	1.70	0.25	2.74	0.75	0.12	0.13	2.28^b^	0.43	38.59	42.54^a^
SEM		0.60	0.27	0.59	0.51	0.13	0.02	0.03	0.02	0.06	0.03	0.01	0.01	0.06	0.02	1.23	0.80
0		59.89	19.75	20.46	19.68	1.41^b^	0.43^a^	1.75	0.27	2.98^a^	0.76	0.14^a^	0.11	2.43	0.46^a^	38.51	40.23
200		60.74	19.70	21.79	19.33	1.92^a^	0.38^b^	1.74	0.24	2.63^b^	0.76	0.11^b^	0.12	2.55	0.42^b^	37.93	39.28
SEM		0.49	0.22	0.48	0.41	0.10	0.01	0.02	0.01	0.04	0.02	0.01	0.01	0.05	0.01	1.00	0.65
Source of variation, *p* value																	
MESO level		0.115	0.054	0.623	0.171	0.011	0.058	0.136	0.813	0.218	0.091	0.304	0.161	0.001	0.869	0.901	0.001
NME		0.232	0.879	0.065	0.554	0.003	0.008	0.807	0.061	0.001	0.932	0.033	0.675	0.131	0.041	0.772	0.271
Interaction		0.871	0.657	0.074	0.266	0.097	0.626	0.287	0.604	0.168	0.082	0.808	0.271	0.554	0.978	0.937	0.076
Regression analysis in responses to MESO level																	
Liner		0.836	0.135	0.985	0.105	0.018	0.038	0.341	0.587	0.592	0.103	0.480	0.199	0.001	0.618	0.711	0.001
Quadratic		0.673	0.259	0.408	0.191	0.568	0.056	0.685	0.551	0.821	0.174	0.303	0.403	0.571	0.954	0.754	0.959

*Note*: Means within each column for each effect with uncommon superscripts (a and b) differ significantly (*p* < 0.05).

Abbreviations: AF, abdominal fat; BF, bursa of Fabricius; GIT, gastrointestinal tract; Giz, gizzard; LI, large intestine; Pan, pancreas; PV, proventriculus; SI, small intestine.

^1^ The values are means of 15, 10 and 5 replicates for enzyme, oil level and interaction effect, respectively.

^2^ The level of dietary metabolizable energy supplied by oil.

^3^ Carcass obtained by feathers, head, feet, skin and viscera removed; frame is the carcass without the breast and legs.

In modern poultry industry bounds, excessive fat is a significant problem (Zhou et al. 2006). To formulate diets that promote reduction in body fat deposition, it is very important to understand the actual energy utilization efficiencies and energy requirements for protein and fat deposition (Sakomura et al. 2005). In the current study, increased MESO content of the diets produced carcasses with higher abdominal fat deposition. This result confirms the effect observed by other researchers (Crespo and Esteve‐Garcia 2001; Vila and Esteve‐Garcia 1996). Moreover, a linear increase in the CL of breast meat by increasing dietary MESO level can be related to the higher meat fat content. To judge the total body fat content, we can utilize abdominal fat pad as a trusted parameter because it is directly tied to total body fat content in avian species (Crespo and Esteve‐Garcia 2001; Fouad and El‐Senousey 2014). How much fat accumulates in the body of an avian relies on the available plasma lipid substrate, originating from the diet or de novo lipogenesis in liver. In the current study, liver relative weight is decreased by increasing dietary MESO level. In agreement with our achievement, Sanz et al. (2000) reported that the inclusion of MESO in the diet of broilers led to an inhibition of the fatty acid synthase (FAS) in the liver. When the low‐energy diets are used for broilers, they contain only a small percentage of fat, and the lipids present in the fowl are derived mainly from carbohydrates. Consequently, the amount of fat accumulated in the body depends to a large extent on the activity of the glycolytic system and, in turn, on the carbohydrate content of the diet (Wickramasuriya et al. 2022). Because fatty acid synthesis is coupled to glycolysis, a high dietary intake of carbohydrates may be expected to cause a marked increase in the fat content of the liver. The rate of hepatic lipogenesis is also influenced by the intake of fat but in the opposite direction, there being a depression of fatty acid synthesis when fat is added to the diet (Butler 1976). It is reported that the sources and levels of lipids in poultry diets may affect their body fat deposition (Fouad and El‐Senousey 2014). In the current study, by dietary enzyme supplementation, abdominal fat accumulation increased. This may be due to the improvement of dietary energy value (Zarghi, Golian, Kermanshahi et al. 2010). Considering the body fat issue, the level of dietary energy is of importance to modify its deposition. Additionally, it is accepted that inhibiting the absorption of dietary fat, which is the consequence of feeding diets with high NSP levels, may reduce abdominal fat deposition by decreasing the size and/or number of abdominal adipose cells (Fouad and El‐Senousey 2014).

In the birds fed the NME‐supplemented diet, the significant decrease in the weight of empty gizzard and small and large intestines is maybe due to the damping function of these parts because of an increase in water‐soluble NSP and a subsequent increase in digesta viscosity. This implies a feedback mechanism in gut motility and thus causes an increase in the size of this organ. Thus, this may lead to an increase in the size of the GIT organs. When the need for enzymes is rising, this increase in the size of GIT organs happens, possibly as an adaptive response (Brenes et al. 1993). To possibly help to reduce the size of the organ, we could use a wheat‐based diet, which supplies exogenous enzymes, so that subsequently a greater proportion of NSP may be hydrolyzed, and it might satisfy the secretory function of the responding organ and the tract segments. The viscosity of NSPs, which leads to a high water‐holding capacity by intestinal contents, is the main issue associated with increasing NSP‐containing ingredients in poultry diets (Józefiak et al. 2007). The reduction of the intestinal chyme viscosity is suggested to be one of the main functions of exogenous enzymes (Teymouri, Zarghi, and Golian 2018).

### 3.3 Tibia Bone Mechanical Properties and Mineral Contents

The main and interactive effects of dietary MESO levels and NME supplementation on tibia bone characteristics, mineral concentration and strength of birds, which were sampled on Day 28, are given in Table 4. The effects of dietary MESO level and interactions of dietary MESO levels and NME supplementation on tibia bone characteristics, mineral concentration and strength were not significant. Enzyme cocktail supplementation led to a significant (*p* < 0.05) increase in bone weight, SG and breaking strength (*p* < 0.05). By supplementing the diet with 200 mg/kg NME, the tibia bone weight, SG and N (force that is required to reach the structural failure) are significantly enhanced.

**TABLE 4:** Effect of dietary non‐starch polysaccharide multi‐enzyme (NME) supplementation and levels of metabolizable energy supply by soy oil (MESO) on tibia bone characteristic and jejunum histology of broiler chickens fed wheat‐based diet (28 days of age)^1^.

		Tibia bone characteristic					Tibia bone mineral concentration					Tibia bone strength			Jejunum histology			
NME, mg/kg	MESO level^2^, %	Weight, g	length, mm	Diameter, mm	Ash, %	SG, g/cm3	Mg, %	Mn, ppm	Zn, ppm	Ca, %	P, %	EB, MJ	BS, N	S, N/mm	VH, µm	CD, µm	VH/CD	MT, µm
0	0	2.49^ab^	71.40	6.02	41.09	1.023	0.37	4.7	240	15.58	7.29	0.010	50	94	1086	240	4.52	199^ab^
	6	2.43^ab^	71.44	5.93	43.22	1.042	0.39	4.5	241	16.31	7.62	0.009	41	99	1003	215	4.69	248^a^
	12	2.36^b^	72.12	5.82	43.10	1.050	0.40	4.8	250	16.53	7.98	0.011	49	92	1014	213	4.82	230^a^
200	0	2.30^b^	71.67	5.94	41.95	1.078	0.37	4.6	262	16.12	7.59	0.020	63	105	1103	238	4.64	168^b^
	6	2.68a^b^	71.48	5.82	40.43	1.051	0.36	4.7	253	16.63	7.40	0.011	51	100	1047	217	4.84	204^ab^
	12	2.89^a^	74.69	6.44	41.80	1.058	0.37	4.8	252	15.71	7.47	0.013	71	115	1058	217	4.91	221^ab^
SEM		0.11	1.43	0.19	0.95	0.015	0.01	0.24	9.65	0.52	0.28	0.002	6.36	6.90	59.93	10.45	0.34	12.52
	0	2.40	71.54	5.98	41.52	1.051	0.37	4.7	251	15.85	7.44	0.015	57	100	1095	239	4.58	184^b^
	6	2.55	71.46	5.88	41.83	1.047	0.38	4.6	247	15.97	7.51	0.010	46	99	1025	216	4.75	226^a^
	12	2.62	73.41	6.13	42.45	1.054	0.39	4.8	251	16.12	7.72	0.012	60	104	1036	215	4.85	225^a^
SEM		0.08	1.01	0.13	0.67	0.011	0.01	0.17	6.82	0.37	0.20	0.002	4.49	4.87	42.38	7.39	0.24	8.85
0		2.42^b^	71.65	5.92	42.47	1.039	0.39	4.7	244	16.14	7.63	0.010^b^	47^b^	95	1035	223	4.67	226^a^
200		2.62^a^	72.61	6.07	41.39	1.062	0.37	4.7	256	15.82	7.49	0.015^a^	62^a^	107	1069	224	4.78	198^b^
SEM		0.06	0.82	0.11	0.55	0.009	0.01	0.14	5.57	0.30	0.16	0.001	3.67	3.98	36.60	6.04	0.19	7.23
Source of variation, *p* value																		
MESO level		0.146	0.337	0.420	0.619	0.877	0.318	0.664	0.878	0.875	0.601	0.141	0.117	0.793	0.481	0.064	0.709	0.008
NME		0.047	0.425	0.363	0.192	0.081	0.164	0.822	0.137	0.466	0.558	0.027	0.013	0.061	0.492	0.898	0.683	0.018
Interaction		0.020	0.631	0.140	0.199	0.251	0.443	0.853	0.588	0.394	0.383	0.245	0.635	0.312	0.965	0.954	0.995	0.409
Regression analysis in responses to MESO level																		
Liner		0.146	0.190	0.472	0.380	0.832	0.157	0.546	0.960	0.607	0.331	0.270	0.117	0.627	0.280	0.010	0.682	0.031
Quadratic		0.743	0.405	0.322	0.862	0.690	0.701	0.434	0.626	0.969	0.761	0.186	0.093	0.731	0.403	0.146	0.868	0.116

*Note*: Means within each column for each effect with uncommon superscripts (a and b) differ significantly (*p* < 0.05).

Abbreviations: BS, breaking strength; CD, crypt depth; EB, energy between; MT, muscular thickness (µm); S, stiffness; SG, specific gravity; VH, villus height.

^1^ The values are means of 15, 10 and 5 replicates for enzyme, oil level and interaction effect, respectively.

^2^ The level of dietary metabolizable energy supplied by oil.

Leg disorders in broiler chickens are paramount welfare and economic issues of the poultry industry. Leg deformity in broilers due to genetic, nutritional or growth factors can lead to a decrease in feed consumption and body weight. Therefore, feed optimization may be a strategy to reduce the severity of leg lesions in broilers (Williams et al. 2000). In agreement with the current study, it has been shown that bone quality parameters were improved when enzymes were supplemented to nutrient‐diluted diets (Lalpanmawia et al. 2014; Leyva‐Jimenez et al. 2019; Walters et al. 2019). The possible reason for these effects with the use of multi‐enzyme supplement is that xylanase reduces digestive viscosity and enables better absorption of nutrients, and also helps to improve the breaking force of broilers' bones and the percentage of bone ash (Bauer 2023). Enzyme supplementation can help release more nutrients from feed for better bone growth and development in broilers (Abu‐Tayyeb et al. 2019). In agreement with the current study, the use of different sources of oil in the broiler chicken's diet did not have a significant effect on bone quality traits, except for calcium and phosphorus concentration (Abdulla et al. 2017). Similarly, other researchers’ (Lau et al. 2013; Lau et al. 2010; Watkins et al. 2000) observations confirm consistent and reproducible beneficial effects of omega‐3 fatty acids on bone metabolism and bone/joint diseases.

### 3.4 Intestinal Morphology

The main and interactive effects of dietary MESO levels and NME supplementation on morphological measurements of broiler chicken jejunum, which were sampled at 28 days of age, are given in Table 4. The effect of NME addition to the diet on all of the jejunal morphological measurements was not significant, except for an effect on muscular thickness (*p* < 0.03). By increasing NME in diet, the jejunal muscular thickness significantly reduced (*p* < 0.01). With increasing MESO levels in diet, the CD reduced (*p* < 0.01), and muscular thickness increased (*p* < 0.02). The effect of interaction between dietary MESO level with NME supplementation on all morphological traits was not significant. The higher NSP in wheat can increase digesta viscosity and reduce enzyme–nutrient binding and their subsequent substrates, which can lead to significant modifications of the structure and function of intestine (Wang et al. 2005). Additionally, to adapt to these changes, the activities of the intestinal secretory mechanisms may be enhanced (Brenes et al. 1993).

### 3.5 Immune Responses

The antibody titre responses to SRBC inoculation, at 24 and 31 days of age, of broiler chickens fed wheat‐based diet with different dietary levels of MESO and supplemented with/without NME, are shown in Table 5. Dietary MESO levels significantly and linearly (*p* < 0.05) improved the primary immune response to SRBC injection. Serum IgT and IgM concentration significantly increased in response to the SRBC inoculation, but in the second stage it is not significant. The dietary NME supplementation and the interaction effects of dietary NME supplementation and MESO levels had no significant effect on immune response to SRBC inoculation. Some fatty acids are essential for poultry because birds are unable to synthesize or convert one fatty acid to another fatty acid of the same type (Salari, Golian, and Hassanabadi 2024). Deficiency of these essential fatty acids may lead to impaired development and immune function. It has been reported that the deficiency of linoleic acid in broilers can delay growth and reduce resistance to diseases (Nayebpor, Hashemi, and Farhomand 2007). It has been reported that the oil source can be effective on the antibody titre in response to the inoculation of sheep blood cells (Fritsche, Cassity, and Huang 1991). As presented in Table 3, the bursa of Fabricius weight as a percentage of LBW at Day 38 of age decreased by NME supplementation and/or 6% and 12% supply dietary metabolizable energy by MESO. According to the present study, it was reported that by using a corn‐wheat (10%–30%)–soybean meal base diet, birds fed with soybean oil had lower thymus weights (Allahyari‐Bake and Jahanian 2017).

**TABLE 5:** Effect of dietary non‐starch polysaccharide multi‐enzyme (NME) supplementation and levels of metabolizable energy supply by soy oil (MESO) on antibody titres responses to sheep red blood cell (SRBC) inoculation at 24 and 31 days of age of broiler chickens fed wheat‐based diet.^1^

NME, mg/kg	MESO level^2^, %	Seven days after the first injection			Seven days after the second injection		
		IgT^3^	IgG^3^	IgM^3^	IgT^3^	IgG^3^	IgM^3^
0	0	3.00^b^	1.00	2.00^b^	4.25	1.25	3.00
	6	4.74^a^	1.25	3.50^a^	3.50	1.50	2.00
	12	4.00^ab^	1.00	3.00^ab^	4.25	2.25	2.00
200	0	3.25^ab^	1.00	2.50^ab^	5.00	2.00	3.00
	6	4.25^ab^	1.00	3.25^ab^	5.50	3.25	2.25
	12	4.25^ab^	1.00	3.25^ab^	4.50	2.00	2.50
SEM		0.59	0.10	0.59	0.58	0.49	0.49
							
	0	3.13	1.00	2.13	4.63	1.63	3.00
	6	4.50	1.13	3.38	4.50	2.38	2.13
	12	4.13	1.00	3.13	4.38	2.13	2.25
SEM		0.42	0.07	0.42	0.41	0.35	0.35
							
0		3.92	1.08	2.83	4.00	1.67	2.33
200		3.92	1.00	2.92	5.00	2.42	2.58
SEM		0.34	0.06	0.34	0.33	0.28	0.27
Source of variation, *p* value							
MESO level		0.045	0.387	0.035	0.913	0.319	0.191
NME		0.965	0.331	0.865	0.055	0.077	0.545
Interaction		0.766	0.387	0.889	0.325	0.153	0.882
Regression analysis in responses to MESO level							
Liner		0.025	0.189	0.024	0.697	0.366	0.136
Quadratic		0.182	0.172	0.132	0.672	0.298	0.382

*Note*: Means within each column for each effect with uncommon superscripts (a and b) differ significantly (*p* < 0.05).

^1^ The values are means of 15, 10 and 5 replicates for enzyme, oil level and interaction effect, respectively.

^2^ The level of dietary metabolizable energy supplied by oil.

^3^ Data represent means of log2 of the reciprocal of the last dilution exhibiting agglutination. IgT, total Ig, IgG (2‐mercaptoethanol‐resistant Ig) and IgM (2‐mercaptoethanol‐sensitive Ig) antibody titres.

### 3.6 Blood Metabolites

It was not observed a significant difference in blood metabolites between birds fed diet with different levels of MESO and/or supplemented by NME measured at 38 days of age (data not reported). Similar to our study, it was reported that adding a xylanase supplement to a wheat‐based diet did not have any effect on the blood profile (Hosseini et al. 2018; Wickramasuriya et al. 2022). In the present study, the effects of MESO and NME supplementation on liver functional enzymes (ALT, AST and ALP), were not significant. Measurement of ALT and AST activities indicates liver damage in broilers and is therefore a valuable tool to determine the safe inclusion of feed additives, as diets may affect serum enzyme activity (Ghavi et al. 2020).

## 4 Conclusion

The results obtained from the current experiment suggest that dietary supplementation by VO plays an important role in lipid metabolism and body fat deposition. In the starter period of rearing broiler chickens, the bird's physiological limitations in lipid digestion and absorption prevent the practical effect of dietary oil levels, but during the later ages, increasing oil levels led to improved growth performance. However, it led to enhanced abdominal fat and CL. Due to the complex structure of wheat grains, it has been shown that NME supplementation can improve growth performance, digestive tract and bone strength. Lower liver relative weight of birds fed diet supplying 12% of the ME requirement through soy oil suggests that soy oil could cause an inhibition of lipogenesis.

## Author Contributions

**Avisa Akhavan Khaleghi**: investigation, methodology. **Abolghasem Golian**: funding acquisition, conceptualization, project administration. **Hassan Nasiri Moghaddam**: funding acquisition, project administration. **Heydar Zarghi**: conceptualization, investigation, writing–original draft, methodology, writing–review and editing, formal analysis, supervision, data curation.

## Disclosure

The authors declare that all of the authors listed on the manuscript were employed at an academic or research institution where research or education is the primary function of the entity. Moreover, this manuscript is independently submitted by the authors.

## Ethics Statement

The authors confirm that the ethical policies of the journal, as noted in the journal's author guidelines page, have been adhered to and the appropriate ethical review committee approval has been received. The authors confirm that they have followed EU standards for the protection of animals used for scientific purposes and feed legislation.

## Conflicts of Interest

The authors declare no conflicts of interest.

### Peer Review

The peer review history for this article is available at https://publons.com/publon/10.1002/vms3.70246.

## Data Availability

The data that support the findings of this study are available from the corresponding author upon reasonable request.

## References

[vms370246-bib-0001] Abdulla, N. , T. Loh , H. Akit , et al. 2017. “Effects of Dietary Oil Sources, Calcium and Phosphorus Levels on Growth Performance, Carcass Characteristics and Bone Quality of Broiler Chickens.” Journal of Applied Animal Research 45, no. 1: 423–429. 10.1080/09712119.2016.1206903.

[vms370246-bib-0002] Abu‐Tayyeb, M. , I. Jahan , M. Hossain , M. Hossain , N. Akter , and B. Nath . 2019. “The Responses of Exogenous Enzymes (Multi‐Enzymes) on the Productivity and Stage of Production of Broilers Fed Vegetable‐Sourced Diets.” International Journal of Poultry Science 18, no. 11: 515–522. 10.3923/ijps.2019.515.522.

[vms370246-bib-0003] Allahdo, P. , J. Ghodraty , H. Zarghi , Z. Saadatfar , H. Kermanshahi , and M. Edalatian Dovom . 2018. “Effect of Probiotic and Vinegar on Growth Performance, Meat Yields, Immune Responses, and Small Intestine Morphology of Broiler Chickens.” Italian Journal of Animal Science 17, no. 3: 675–685. 10.1080/1828051X.2018.1424570.

[vms370246-bib-0060] Allahyari‐Bake, S. , and R. Jahanian . 2017. “Effects of Dietary fat Source and Supplemental Lysophosphatidylcholine on Performance, Immune Responses, and Ileal Nutrient Digestibility In Broilers fed Corn/Soybean Meal‐or Corn/Wheat/Soybean Meal‐Based Diets.” Poultry Science 96, no. 5: 1149‐1158. 10.3382/ps/pew330 27697931

[vms370246-bib-0004] Amer, S. A. , A. A‐Nasser , H. S. Al‐Khalaifah , et al. 2020. “Effect of Dietary Medium‐Chain α‐Monoglycerides on the Growth Performance, Intestinal Histomorphology, Amino Acid Digestibility, and Broiler Chickens' Blood Biochemical Parameters.” Animals 11, no. 1: 57. 10.3390/ani11010057. 33396850 PMC7823994

[vms370246-bib-0062] AOAC . 2002. Official Methods of Analysis of AOAC International. 17th ed.. Association of Official Analytical Chemists.

[vms370246-bib-0005] Attia, Y. , N. Addeo , E. Abd Al‐Hamid , and F. Bovera . 2019. “Effects of Phytase Supplementation to Diets With or Without Zinc Addition on Growth Performance and Zinc Utilization of White Pekin Ducks.” Animals 9, no. 5: 280. 10.3390/ani9050280. 31130648 PMC6562945

[vms370246-bib-0063] Aviagen . 2022. Ross broiler: Nutrition specification. Aviagen.

[vms370246-bib-0007] Azman, M. , V. Konar , and P. Seven . 2004. “Effects of Different Dietary Fat Sources on Growth Performances and Carcass Fatty Acid Composition of Broiler Chickens.” Revue De Médecine Vétérinaire 5: 278–286.

[vms370246-bib-0008] Bauer, M. 2023. “The Effects of Exogenous Enzyme Supplementation on the Performance, Bone Quality, and Nutrient Utilization of Broiler Chickens.” Master of Science, University of Kentucky.

[vms370246-bib-0009] Brenes, A. , M. Smith , W. Guenter , and R. Marquardt . 1993. “Effect of Enzyme Supplementation on the Performance and Digestive Tract Size of Broiler Chickens Fed Wheat‐and Barley‐Based Diets.” Poultry Science 72, no. 9: 1731–1739. 10.3382/ps.0721731. 8234133

[vms370246-bib-0010] Butler, E. J. 1976. “Fatty Liver Diseases in the Domestic Fowl—A Review.” Avian Pathology 5, no. 1: 1–14. 10.1080/03079457608418164. 18777323

[vms370246-bib-0011] Chizari, A. , and M. Hajiheidary . 2010. “The Effects of Market Factors and Government Policies on Maize Marketing in Iran.” African Journal of Agricultural Research 5, no. 12: 1351–1359. 10.5897/AJAR09.590.

[vms370246-bib-0012] Cho, S. , H. Lee , I. Chung , et al. 2008. “Effects of Dietary Energy Concentration and Lysine on the Digestible Energy Ratio for Apparent Amino Acid Digestibility in Finishing Barrows.” Asian–Australasian Journal of Animal Sciences 21, no. 2: 232–236.

[vms370246-bib-0013] Crespo, N. , and E. Esteve‐Garcia . 2001. “Dietary Fatty Acid Profile Modifies Abdominal Fat Deposition in Broiler Chickens.” Poultry Science 80, no. 1: 71–78. 10.1093/ps/80.1.71. 11214339

[vms370246-bib-0014] Cufadar, Y. , O. Olgun , and A. Yildiz . 2011. “The Effect of Dietary Calcium Concentration and Particle Size on Performance, Eggshell Quality, Bone Mechanical Properties and Tibia Mineral Contents in Moulted Laying Hens.” British Poultry Science 52, no. 6: 761–768. 10.1080/00071668.2011.641502. 22221242

[vms370246-bib-0015] Ebrahimi, E. , S. Sobhani rad , and H. Zarghi . 2017. “Effect of Triticale Level and Exogenous Enzyme in the Grower Diet on Performance, Gastrointestinal Tract Relative Weight, Jejunal Morphology and Blood Lipids of Japanese Quail (Coturnix *Coturnix japonica*).” Journal of Agricultural Science and Technology 19: 569–580.

[vms370246-bib-0016] Fébel, H. , M. Mezes , T. Pálfy , et al. 2008. “Effect of Dietary Fatty Acid Pattern on Growth, Body Fat Composition and Antioxidant Parameters in Broilers.” Journal of Animal Physiology and Animal Nutrition 92, no. 3: 369–376. 10.1111/j.1439-0396.2008.00803.x. 18477319

[vms370246-bib-0018] Fouad, A. , and H. El‐Senousey . 2014. “Nutritional Factors Affecting Abdominal Fat Deposition in Poultry: A Review.” Asian–Australasian Journal of Animal Sciences 27, no. 7: 1057–1068. 10.5713/ajas.2013.13702. 25050050 PMC4093572

[vms370246-bib-0019] Fritsche, K. , N. Cassity , and S. Huang . 1991. “Effect of Dietary Fat Source on Antibody Production and Lymphocyte Proliferation in Chickens.” Poultry Science 70, no. 3: 611–617. 10.3382/ps.0700611. 2047352

[vms370246-bib-0061] Ghavi, S. , H. Zarghi , and A. Golian . 2020. “Effect of Dietary Digestible Sulphur Amino Acids Level on Growth Performance, Blood Metabolites and Liver Functional Enzymes of Broilers 1–11 Days of Age.” Italian Journal of Animal Science 19, no. 1: 1439–1449. 10.1111/jpn.13214 31559663

[vms370246-bib-0020] Hempe, J. , R. Laukxen , and J. Savage . 1988. “Rapid Determination of Egg Weight and Specific Gravity Using a Computerized Data Collection System.” Poultry Science 67: 902–907. 10.3382/ps.0670902. 3413014

[vms370246-bib-0021] Hossaninejad, S. , H. Zarghi , and A. Golian . 2021. “Effect of Digestible Threonine on Performance, Egg Quality, Blood Metabolites, and Immune Responses in Laying Hens Fed a Wheat‐Based Diet in the Second Cycle.” Italian Journal of Animal Science 20, no. 1: 2134–2146. 10.1080/1828051X.2021.2004248.

[vms370246-bib-0022] Hosseini, S. , R. Nourmohammadi , H. Nazarizadeh , and J. D. Latshaw . 2018. “Effects of Lysolecithin and Xylanase Supplementation on the Growth Performance, Nutrient Digestibility and Lipogenic Gene Expression in Broilers Fed Low‐Energy Wheat‐Based Diets.” Journal of Animal Physiology and Animal Nutrition 102, no. 6: 1564–1573. 10.1111/jpn.12966. 30043541

[vms370246-bib-0023] Józefiak, D. , A. Rutkowski , B. Jensen , and R. Engberg . 2007. “Effects of Dietary Inclusion of Triticale, Rye and Wheat and Xylanase Supplementation on Growth Performance of Broiler Chickens and Fermentation in the Gastrointestinal Tract.” Animal Feed Science and Technology 132, no. 1–2: 79–93. 10.1016/j.anifeedsci.2006.03.011.

[vms370246-bib-0024] Katongole, J. , and B. March . 1980. “Fat Utilization in Relation to Intestinal Fatty Acid Binding Protein and Bile Salts in Chicks of Different Ages and Different Genetic Sources.” Poultry Science 59: 819–827. 10.3382/ps.0590819. 7375429

[vms370246-bib-0025] Kolakshyapati, M. , R. Flavel , T. Sibanda , D. Schneider , M. Welch , and I. Ruhnke . 2019. “Various Bone Parameters Are Positively Correlated With Hen Body Weight While Range Access Has no Beneficial Effect on Tibia Health of Free‐Range Layers.” Poultry Science 98, no. 12: 6241–6250. 10.3382/ps/pez487. PMC8913749 31504903

[vms370246-bib-0026] Lalpanmawia, H. , A. Elangovan , M. Sridhar , D. Shet , S. Ajith , and D. Pal . 2014. “Efficacy of Phytase on Growth Performance, Nutrient Utilization and Bone Mineralization in Broiler Chicken.” Animal Feed Science and Technology 192: 81–89. 10.1016/j.anifeedsci.2014.03.004.

[vms370246-bib-0027] Lau, B. , D. Cohen , W. Ward , and D. Ma . 2013. “Investigating the Role of Polyunsaturated Fatty Acids in Bone Development Using Animal Models.” Molecules (Basel, Switzerland) 18, no. 11: 14203–14227. 10.3390/molecules181114203. 24248147 PMC6270577

[vms370246-bib-0028] Lau, B. , V. Fajardo , L. McMeekin , et al. 2010. “Influence of High‐Fat Diet From Differential Dietary Sources on Bone Mineral Density, Bone Strength, and Bone Fatty Acid Composition in Rats.” Applied Physiology, Nutrition, and Metabolism 35, no. 5: 598–606. 10.1139/H10-052. 20962915

[vms370246-bib-0029] Leyva‐Jimenez, H. , A. Alsadwi , K. Gardner , E. Voltura , and C. Bailey . 2019. “Evaluation of High Dietary Phytase Supplementation on Performance, Bone Mineralization, and Apparent Ileal Digestible Energy of Growing Broilers.” Poultry Science 98, no. 2: 811–819. 10.3382/ps/pey389. 30169714

[vms370246-bib-0030] Mirshekar, R. , B. Dastar , B. Shabanpour , and S. Hassani . 2013. “Effect of Dietary Nutrient Density and Vitamin Premix Withdrawal on Performance and Meat Quality of Broiler Chickens.” Journal of the Science of Food and Agriculture 93, no. 12: 2979–2985. 10.1002/jsfa.6127. 23495000

[vms370246-bib-0031] Muszyński, S. , E. Tomaszewska , P. Dobrowolski , et al. 2018. “Analysis of Bone Osteometry, Mineralization, Mechanical and Histomorphometrical Properties of Tibiotarsus in Broiler Chickens Demonstrates a Influence of Dietary Chickpea Seeds (*Cicer arietinum* L.) Inclusion as a Primary Protein Source.” PLoS ONE 13, no. 12: e0208921. 10.1371/journal.pone.0208921. 30533027 PMC6289425

[vms370246-bib-0032] Nayebpor, M. , A. Hashemi , and P. Farhomand . 2007. “Influence of Soybean Oil on Growth Performance, Carcass Properties, Abdominal Fat Deposition and Humoral Immune Response in Male Broiler Chickens.” Journal of Animal and Veterinary Advances 6, no. 11: 1317–1322.

[vms370246-bib-0033] Nikbakhtzade, M. , H. Zarghi , and A. Golian . 2024. “Effects of Finisher Diet Nutrients Density and Slaughter Age on Energy and Protein Efficiency, Productive and Economic Performance and Meat Quality of Broilers.” Veterinary Medicine and Science 10, no. 4: e1493. 10.1002/vms3.1493. 38923740 PMC11196377

[vms370246-bib-0034] NRC . 1994. Nutrient Requirements of Poultry, 9th rev. ed. The National Academies Press.

[vms370246-bib-0035] Ravindran, V. , P. Tancharoenrat , F. Zaefarian , and G. Ravindran . 2016. “Fats in Poultry Nutrition: Digestive Physiology and Factors Influencing Their Utilisation.” Animal Feed Science and Technology 213: 1–21. 10.1016/j.anifeedsci.2016.01.012.

[vms370246-bib-0036] Richard, O. , R. Kellems , and D. Church . 2010. Livestock Feeds and Feeding, 5 illustrated ed.. Prentice Hall.

[vms370246-bib-0037] Sakomura, N. , F. Longo , E. Oviedo‐Rondon , C. Boa‐Viagem , and A. Ferraudo . 2005. “Modeling Energy Utilization and Growth Parameter Description for Broiler Chickens.” Poultry Science 84, no. 9: 1363–1369. 10.1093/ps/84.9.1363. 16206556

[vms370246-bib-0038] Salari, A. , A. Golian , and A. Hassanabadi . 2024. “Effect of Dietary Lysophospholipid Supplementation on Growth Performance, Serum Lipids, Small Intestine Morphology and Caeca Microflora in Broiler Chickens.” Veterinary Medicine and Science 10, no. 1: e1303. 10.1002/vms3.1303. 38109278 PMC10766047

[vms370246-bib-0039] Sanz, M. , C. Lopez‐Bote , D. Menoyo , and J. Bautista . 2000. “Abdominal Fat Deposition and Fatty Acid Synthesis Are Lower and β‐Oxidation Is Higher in Broiler Chickens Fed Diets Containing Unsaturated Rather Than Saturated Fat.” Journal of Nutrition 130: 3034–3037. 10.1093/jn/130.12.3034. 11110864

[vms370246-bib-0040] SAS . 2014. SAS User's Guide: Statistics. Version 9.4. SAS Inst. Inc.

[vms370246-bib-0041] Tancharoenrat, P. , V. Ravindran , F. Zaefarian , and G. Ravindran . 2013. “Influence of Age on the Apparent Metabolisable Energy and Total Tract Apparent Fat Digestibility of Different Fat Sources for Broiler Chickens.” Animal Feed Science and Technology 186, no. 3–4: 186–192. 10.1016/j.anifeedsci.2013.10.013.

[vms370246-bib-0042] Tancharoenrat, P. , V. Ravindran , F. Zaefarian , and G. Ravindran . 2014. “Digestion of Fat and Fatty Acids Along the Gastrointestinal Tract of Broiler Chickens.” Poultry Science 93, no. 2: 371–379. 10.3382/ps.2013-03344. 24570459

[vms370246-bib-0043] Teymouri, H. , H. Zarghi , and A. Golian . 2018. “Evaluation of Hull‐Less Barley With or Without Enzyme Cocktail in the Finisher Diets of Broiler Chickens.” Journal of Agricultural Science and Technology 20, no. 3: 469–483.

[vms370246-bib-0044] UFFDA . 1992. User‐Friendly Feed Formulation Done Again. University of Georgia.

[vms370246-bib-0045] Velasco, S. , L. Ortiz , C. Alzueta , A. Rebole , J. Trevino , and M. Rodriguez . 2010. “Effect of Inulin Supplementation and Dietary Fat Source on Performance, Blood Serum Metabolites, Liver Lipids, Abdominal Fat Deposition, and Tissue Fatty Acid Composition in Broiler Chickens.” Poultry Science 89, no. 8: 1651–1662. 10.3382/ps.2010-00687. 20634521

[vms370246-bib-0046] Vila, B. , and E. Esteve‐Garcia . 1996. “Studies on Acid Oils and Fatty Acids for Chickens. I. Influence of Age, Rate of Inclusion and Degree of Saturation on Fat Digestibility and Metabolisable Energy of Acid Oils.” British Poultry Science 37, no. 1: 105–117. 10.1080/00071669608417841. 8833532

[vms370246-bib-0047] Walters, H. , M. Coelho , C. Coufal , and J. Lee . 2019. “Effects of Increasing Phytase Inclusion Levels on Broiler Performance, Nutrient Digestibility, and Bone Mineralization in Low‐Phosphorus Diets.” Journal of Applied Poultry Research 28, no. 4: 1210–1225. 10.3382/japr/pfz087.

[vms370246-bib-0048] Wang, Z. , S. Qiao , W. Lu , and D. Li . 2005. “Effects of Enzyme Supplementation on Performance, Nutrient Digestibility, Gastrointestinal Morphology, and Volatile Fatty Acid Profiles in the Hindgut of Broilers Fed Wheat‐Based Diets.” Poultry Science 84, no. 6: 875–881. 10.1093/ps/84.6.875. 15971523

[vms370246-bib-0049] Watkins, B. , Y. Li , K. Allen , W. Hoffmann , and M. Seifert . 2000. “Dietary Ratio of (n‐6)/(n‐3) Polyunsaturated Fatty Acids Alters the Fatty Acid Composition of Bone Compartments and Biomarkers of Bone Formation in Rats.” Journal of Nutrition 130, no. 9: 2274–2284. 10.1093/jn/130.9.2274. 10958824

[vms370246-bib-0050] Wickramasuriya, S. , S. Macelline , E. Kim , et al. 2022. “Exogenous Emulsifiers and Multi‐Enzyme Combination Improves Growth Performance of the Young Broiler Chickens Fed Low Energy Diets Containing Vegetable Oil.” Animal Bioscience 35, no. 10: 1585–1591. 10.5713/ab.22.0024. 35468275 PMC9449380

[vms370246-bib-0051] Williams, B. , S. Solomon , D. Waddington , B. Thorp , and C. Farquharson . 2000. “Skeletal Development in the Meat‐Type Chicken.” British Poultry Science 41, no. 2: 141–149. 10.1080/713654918. 10890208

[vms370246-bib-0052] Wongsuthavas, S. , C. Yuangklang , S. Wittayakun , et al. 2007. “Dietary Soybean Oil, But Not Krabok Oil, Diminishes Abdominal Fat Deposition in Broiler Chickens.” International Journal of Poultry Science 6, no. 11: 792–795.

[vms370246-bib-0053] Xavier, R. S. , E. Freitas , N. Braz , et al. 2015. “Limestone Particle Sizes and Lighting Regimens on Egg and Bone Quality of Laying Hens.” Pesquisa Agropecuária Brasileira 50: 718–725. 10.1590/S0100-204x2015000800010.

[vms370246-bib-0054] Zarghi, H. , A. Golian , and H. Aghel . 2010. “Effect of Triticale on Performance and Blood Chemistry of Commercial Growing Turkeys.” Global Veterinaria 4, no. 5: 441–446.

[vms370246-bib-0055] Zarghi, H. , A. Golian , A. Hassanabadi , and F. Khaligh . 2022. “Effect of Zinc and Phytase Supplementation on Performance, Immune Response, Digestibility and Intestinal Features in Broilers Fed a Wheat‐Soybean Meal Diet.” Italian Journal of Animal Science 21, no. 1: 430–444. 10.1080/1828051X.2022.2034061.

[vms370246-bib-0056] Zarghi, H. , A. Golian , H. Kermanshahi , A. Raji , and A. Heravi . 2010. “The Effect of Triticale and Enzyme in Finisher Diet on Performance, Gut Morphology and Blood Chemistry of Broiler Chickens.” Journal of Animal and Veterinary Advances 9, no. 10: 2305–2314. 10.3923/JAVAA.2010.2305.2314.

[vms370246-bib-0058] Zhou, H. , N. Deeb , C. Evock‐Clover , C. Ashwell , and S. J. Lamont . 2006. “Genome‐Wide Linkage Analysis to Identify Chromosomal Regions Affecting Phenotypic Traits in the Chicken. II. Body Composition.” Poultry Science 85, no. 10: 1712–1721. 10.1093/ps/85.10.1712. 17012160

